# Graphene Oxide–PEG–Protocatechuic Acid Nanocomposite Formulation with Improved Anticancer Properties

**DOI:** 10.3390/nano8100820

**Published:** 2018-10-11

**Authors:** Bullo Saifullah, Kalaivani Buskaran, Rabia Baby Shaikh, Farahnaz Barahuie, Sharida Fakurazi, Mohd Aris Mohd Moklas, Mohd Zobir Hussein

**Affiliations:** 1 Laboratory for Vaccine and Immunotherapeutics, Institute of Biosciences, Universiti Putra Malaysia (UPM), Serdang 43400, Selangor, Malaysia; bullosaif1@gmail.com (B.S.); vaneey_88@yahoo.com (K.B.); sharida@upm.edu.my (S.F.); 2 Material Synthesis and Characterization Laboratory, Institute of Advanced Technology (ITMA), Universiti Putra Malaysia (UPM), Serdang 43400, Selangor, Malaysia; 3 Department of Human Anatomy Faculty of Medicine and Health Sciences, Universiti Putra Malaysia (UPM), Serdang 43400, Selangor, Malaysia; aris@upm.edu.my; 4 Education Department, Sukkur IBA University, Sukkur 65200, Sindh, Pakistan; rabia.shaikh@iba-sukkur.edu.pk; 5 Faculty of Industry and Mining, University of Sistan and Baluchestan, Zahedan 98167, Sistan and Bauchetan, Iran; farahnaz.barahuie@gmail.com

**Keywords:** graphene oxide, PEG, cancer, drug delivery, protocatechuic acid, liver cancer, colon cancer

## Abstract

The treatment of cancer through chemotherapy is limited by its toxicity to healthy tissues and organs, and its inability to target the cancer site. In this study, we have designed an anticancer nanocomposite delivery system for protocatechuic acid (PCA) using graphene oxide–polyethylene glycol as the nanocarrier, and coated with folic acid (GO–PEG–PCA–FA) for targeting the cancer cells. The designed anticancer delivery system was found to show much better anticancer activity than the free drug PCA against liver cancer HEP-G2 cells and human colon cancer HT-29 cells; at same time, it was found to be less toxic to normal fibroblast 3T3 cells. The folate-coated anticancer delivery system was found to show better activity then the free drug and the uncoated anticancer delivery system. The in vitro release of the PCA was found to be sustained in human physiological pHs, i.e., blood pH 7.4 and intracellular lysosomal pH 4.8. These in vitro findings are highly encouraging for further in vivo evaluation studies.

## 1. Introduction

The emergence of the nanomedicine is one of the most significant advancements in the field of medical science in this century. Nanomedicine has encompassed the advanced medical diagnostics, drug manufacturing, and drug delivery systems. Simultaneous disease detection and therapy or the so-called theranostic studies are also made possible with the advancement of nanomedicine [1,2,3,4,5,6,7,8]. Drug delivery is the key area of nanomedicine, and it is the subject of rapid growth and advancement due to its vital role in the minimization of the adverse side effects of drugs, improvements in therapeutic efficacy, and prolonged bioavailabilty [8,9,10,11,12,13]. Many nanomaterials, namely micelles, dendrimers, liposomes, inorganic metallic nanolayers, carbon nanotubes, polymers, and graphene oxide have been explored for the designing of the nanocarriers for different drugs [5,8,14,15,16,17]. Graphene oxide (GO) is a promising functionalized nanobiomaterial that is being widely applied in drug delivery, biosensing, electronics, photocatalysis, energy storage devices (supercapacitor and batteries), and biomedicine [18,19,20]. Lately, graphene oxide and its modified forms are getting intense attention from the scientific community due to its variety of functional groups, containing surface and various applications in different fields. The interest in the utilisation of GO is due to its biocompatibility and superior physicochemical properties, where GO with its unique structure consist of planar, graphene-like aromatic domains of random sizes interconnected with a six-member ring carbon network functionalized with carboxylic (COOH), carbonyl (C=O), hydroxyl (OH), epoxy, ether, diol, epoxides, and ketones groups. These functional groups enable GO for the further functionalization, conjugation, and/or immobilization of other nanoparticles, polymer fabrication, and the loading of drugs/biomolecules (RNA/DNA, etc.) on its surface [20,21,22,23]. Polyethylene glycol (PEG) is a non-ionic water-soluble polymer that is most widely used in drug delivery, and has also been reported as a phase change material [24,25]. The functionalization of GO with polyethylene glycol (GO–PEG) has been reported to improve the aqueous solution stability, and biocompatibility made it the ideal material to be applied in drug delivery [26,27,28]. GO–PEG has been reported to show an excellent transfection of nucleic acids (plasmid DNA and small interfering RNA) in cells and the administration of conventional drug treatment [22,29]. Furthermore, the efficiency of transfection can be improved by irradiation with a low-power infrared laser [30]. Protocatechuic acid (PCA) (3,4-dihydroxybenzoic acid) is a phenolic compound that is found in several medicinal plants, namely St John’s wort (*Hypericum perforatum* L.), Sudan mallow (*Hibiscus sabdariffa* L.), and Japanese ginkgo (*Ginkgo biloba* L.) [31,32,33]. The previous studies have shown that protocatechuic acid possesses antioxidant properties, and extraordinary pharmacological activities such as antitumor, anticancer, antimutagenic, antibacterial, antigenotoxic, and excellent anti-inflammatory [34,35,36,37]. Furthermore, PCA has been reported to cause significant apoptotic effects in the treatment of various types of cancer cells, namely in the liver, cervix, breast, lung, and leukemia [38]. The folate receptor that is present on the cell surface is overexpressed on the vast majority of cancer tissues, and contrarily, its expression is limited in healthy tissues and organs [39]. Folate receptors are significantly overexpressed in ovarian, epithelial, breast, cervical, lung, kidney, colorectal, and brain tumors [40,41]. The folate receptors have limited expression in the kidneys, lungs, placenta, and choroid plexus [40]. The folate receptor is a cell surface glycoprotein receptor that is able to bind to folate with high affinity and mediate the unidirectional transport of folate into cells. Folate has been used for targeting cancers and tumors, as folate receptors are highly expressed on the surface of many types of tumors [42]. The high affinity of the folate receptor that is preferentially expressed in cancer cells is rarely expressed in normal cells. This has allowed the development of the targeted delivery of anticancer drugs at the cancer at the tumor sites to maximize anticancer efficacy with minimizing side effects to healthy tissues [42,43,44,45]. The folate targeting of cancerous cells has been reported recently in many studies; e.g., L. Xing et al. 2018 used folate targeting for ovary cancer cells, and found that folate receptor alpha (FRα) is overexpressed in ovary cancer cells [42]. In other study, Jun Ai et al. 2018 utilized folic acid and found it to be a better cancer targeting agent, and Ana I et al. 2018 wrote a comprehensive review on folic acid as the directing agent of cancer cells in nanoformulations [46,47]. Since GO has a layered structure with a large surface area, this renders it suitable for the further loading of active agents. Recently, there have been few studies that have utilized the GO–PEG–folic acid coating strategy to target tumors [44,45,48].

In this study, we have designed an anticancer nanocomposite formulation using GO–PEG as the nanocarrier loaded with anticancer drug PCA, and coated it with folic acid (FA) for the active targeting of the different cancer cells.

## 2. Results

### 2.1. X-ray Diffraction Analysis

**Figure 1:**
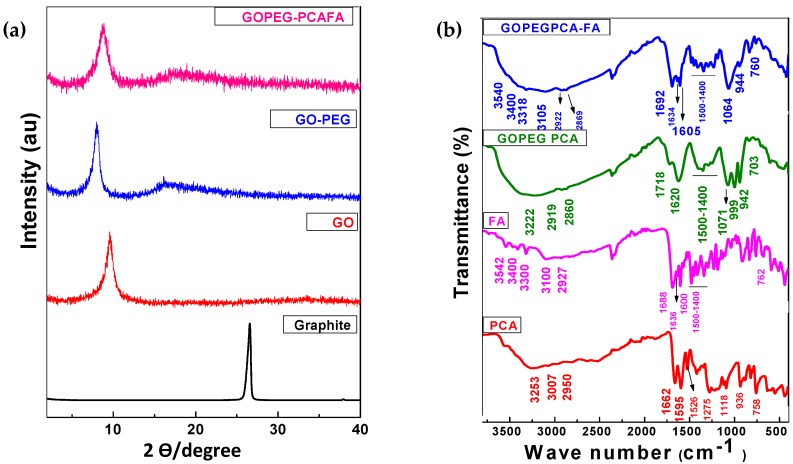
(**a**) XRD diffraction patterns of graphene oxide (GO), GO–polyethylene glycol (PEG), and GO–PEG–protocatechuic acid (PCA)–folic acid (FA) nanocomposite; (**b**) shows the Fourier transformed infrared (FTIR) spectra of free drug PCA, folic acid (FA), nanocomposite GO–PEG–PCA, and the FA-coated nanocomposite GO–PEG–PCA–FA.

Figure 1a shows the X-ray diffraction (XRD) patterns of graphite, graphene oxide (GO), graphene oxide–polyethylene glycol (GO–PEG), and the folic acid (FA)-coated anticancer (GO–PEG–PCA–FA) nanocomposite. Graphite showed the characteristic sharp peak at 2θ degree at 26.1°, corresponding to the diffraction of the (002) plane with the basal spacing of about 3.4 Å [12]. This characteristic graphite peak disappeared in the XRD diffraction patterns of GO, and a new GO characteristic peak appeared at about 2θ = 10.3° with the basal spacing of 8.5 Å. The increase in the basal spacing from 3.4 Å (graphite) to 8.5 Å (GO) can be attributed to the insertion of oxygenated functional groups, namely carboxylic acid, hydroxyl groups, and epoxides between the GO planes [12,49,50]. The appearance of a GO characteristic peak at 2θ = 10.20° with the disappearance of a graphite peak at 26.1° with an increase in the basal spacing strongly indicated the successful formation of GO [17,51]. PEG has been reported to show its two characteristic major sharp intense peaks at 2θ = 19.21° and 23.32°, and the free drug PCA has been reported to show the sharp peaks at about 2θ = 18° [5,52,53]. The XRD patterns for the nanocarrier GO–PEG and the anticancer GO–PEG–PCA–FA nanocomposite showed a small hub between 2θ = 15°–25°, which can be attributed to the presence of PEG and the loaded-drug, PCA. This compliments the successful formation of the GO–PEG nanocarrier and the anticancer nanocomposite GO–PEG–PCA–FA.

### 2.2. Fourier Transformed Infrared Spectroscopic Analysis

Figure 1b shows the Fourier transformed infrared (FTIR) spectra of free drug PCA, free folic acid (FA), anticancer nanocomposite GO–PEG–PCA, and the folic acid-loaded anticancer GO–PEG–PCA–FA nanoparticles. The FTIR spectrum of free drug PCA showed the typical characteristics of functional groups bands, namely a hydroxyl band (3253 cm^−1^), aromatic phenyl C–H bands (3007 cm^−1^ and 2950 cm^−1^), a carbonyl (C=O) band 1662 cm^−1^, aromatic (C=C) bands at 1595 cm^−1^, and the group of bands between 1500–1400 cm^−1^, the C–O band at 936 cm^−1^ and the C–H bending band at 758 cm^−1^ [5]. Folic acid showed the characteristic peaks of the N–H stretching bands at 3542 cm^−1^ and 3400 cm^−1^, the O–H band at 3300 cm^−1^, the C–H bands at 3100 cm^−1^ and 2927 cm^−1^, the carbonyl (C=O) band of carboxylic acid at 1688 cm^−1^, the carbonyl (C=O) of amide at 1636 cm^−1^, the group of bands due to the benzene ring C=C between 1400–1500 cm^−1^ and N–H bending vibration at 762 cm^−1^ [54,55]. The anticancer nanocomposite GO–PEG–PCA showed that the bands belongs to PCA, and with the addition of the ether functional group, C–O bands appeared at 1071 cm^−1^ [52]. The functional group bands for GO such as carbonyl, carboxylic acid, ether (epoxides), and the benzene ring are almost the same as those present in PCA and ether in PEG, as previously reported [12,17]. The FTIR spectrum of the anticancer folic acid-coated nanocomposite (GO–PEG–PCA–FA) showed the functional group bands features of PCA, GO–PEG, and FA, with slight shifts in band positions, which confirms the successful formation of the nanocomposite (GO–PEG–PCA–FA). Table 1 shows the details for all of the functional groups bands that are present in PCA, FA, GO–PEG–CA, and folic acid-coated nanocomposites.

### 2.3. HPLC Analysis for Quantification Drug Loading

High-performance liquid chromatography (HPLC) analysis is the most accurate method that is used for the quantification of drug loading in nanocomposites. The percentage loading of PCA in the nanocomposite GO–PEG–PCA–FA was determined through using the previously developed method with slight modification [56]. The calibration curve was constructed using different standard concentrations of PCA, e.g., 10 ppm, 20 ppm, 30 ppm, 40 ppm, 50 ppm, 100 ppm, 150 ppm, 200 ppm, and 250 ppm. The *r*^2^ of the calibration curve was found to be 0.9934, from which the PCA loading in nanocomposite GO–PEG–PCA–FA was found to be 36.08%. 

### 2.4. High-Resolution Transmission Electron Microscopy (HR-TEM)

**Figure 2:**
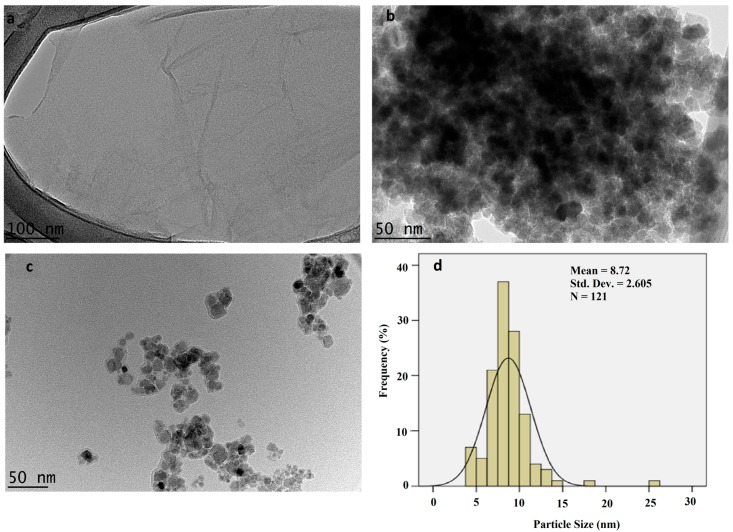

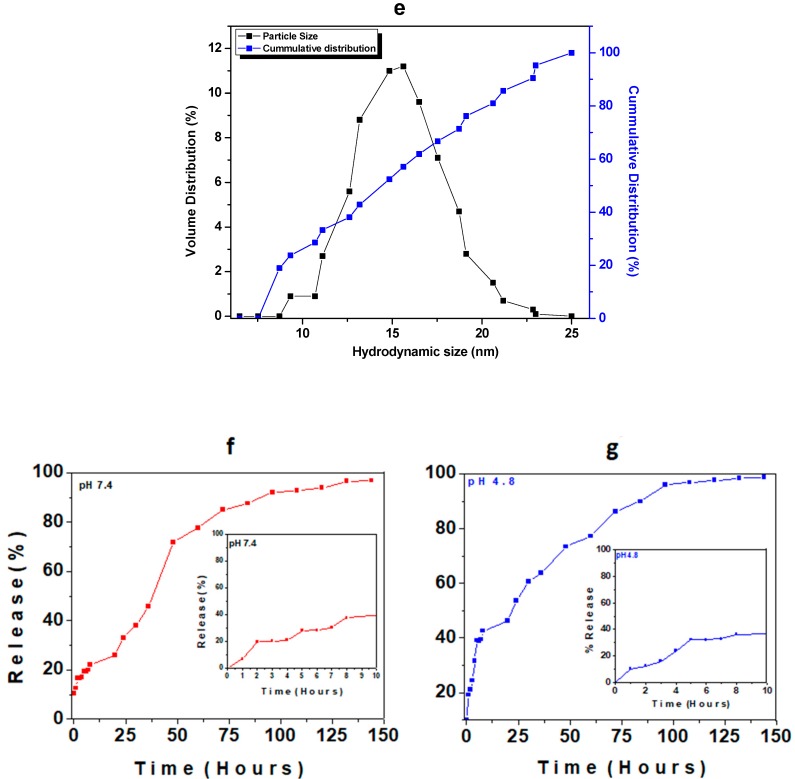
(**a**) High-resolution transmission electron micrographs of GO; (**b**) GO–PEG; and (**c**) GO–PEG–PCA–FA nanocomposite and (**d**) particle size distribution of GO–PEG–PCA–FA nanocomposite; (**e**) particles size distribution determined using dynamic light scattering (DLS); (**f**,**g**) shows the in vitro of PCA from the anticancer nanocomposite GO–PEG–PCA–FA.

The structural and morphological features of GO, GO–PEG, and the GO–PEG–PCA–FA nanocomposites were analyzed using high-resolution transmission electron microscopy (HR-TEM). Figure 2a–d shows the HR-TEM micrographs of GO (a), the empty nanocarrier GO–PEG (b), and the folic acid-coated nanocomposite (GO–PEG–PCA–FA) (c). The HR-TEM image of GO revealed its straight sheet-like shape Figure 2a, the empty nanocarrier GO–PEG Figure 2b revealed that there is the clear fabrication of PEG with GO, as the shape and morphology is more similar to the particulate type (agglomerated particulate), and unlike GO, it is a straight sheet [57]. On the other hand, the shape of GO–PEG–PCA–FA was found to have a particle-type shape with irregular morphology, as shown in Figure 2c. The Figure 2d shows the particle size distribution of nanocomposite GO–PEG–PCA–FA calculated from its HR-TEM using (UTHSCSA Image Tool for Windows Version 3.00) image processing software by randomly selecting 121 particles (N). The particle size distribution was found to be between 4–30 nm, and the average particle size was found to be 8.72 nm.

### 2.5. DLS Analysis

The dynamic light scattering (DLS) technique was used for the analysis of the particle size distribution of the anticancer nanocomposite GO–PEG–PCA–FA using a Zetasizer. The sample was sonicated for 20 min after dispersing it in deionized water, and then analyzed. Figure 2e shows the relative and cumulative particle size distributions. The anticancer nanocomposite GO–PEG–PCA–FA was found to have a narrow size distribution between 8–25 nm, with more than 50% of the particles having a size of 15 nm or less.

### 2.6. In Vitro Release Study

The release study of PCA from the GO–PEG–PCA–FA nanocomposite was conducted in phosphate-buffered saline (PBS) solutions of pH 7.4, which mimic the human blood pH, and pH 4.8, which mimics the intracellular lysosomal pH. The sample was put in a thermostat at 37 °C (human body temperature) with constant shaking, and an aliquot was taken out at different time periods and replaced with the same volume of the new buffer solution. For the first eight hours, the sample was taken with a gap of one hour (1 h, 2 h, 3 h, 4 h, 5 h, 6 h, 7 h, and 8 h) and then, this sequence gap was increased to the following order: 20 h, 24 h, 30 h, 36 h, 48 h, 60 h, 72 h, 84 h, 96 h, 108 h, 120 h, 132 h, and 144 h. Figure 2f (inset) shows the initial first eight hours of PCA release and the main Figure 1h shows the complete release for both cases of different pHs. Figure 2f shows the release profile of PCA in PBS of pH 7.4, and Figure 2g shows the release profile of PCA in PBS of pH 4.8.

For the in vitro release of PBS at pH 7.4, the first 8 h of PCA release was found to be about 40%, followed by extremely sustained release for up to 100 h (90% release), and the complete release took about 140 h. In pH 4.8, about 35% of the PCA was released in the first eight hours, and complete release took about 140 h also. Although the release profile of PCA from nanocomposite GO–PEG–PCA–FA was highly sustained under both physiological conditions, the release was relatively more sustained in the pH 7.4 solution, as it took about 140 h for most of the PCA to be released compared to 100 h in the pH 4.8 solution. The release of PCA can occur because of the ion exchange in a PBS solution of pH 7.4, and by withering/ion exchange in an acid PBS solution of pH 4.8.

### 2.7. Cytotoxicity Studies on Normal Fibroblast (3T3) Cells

**Figure 3:**
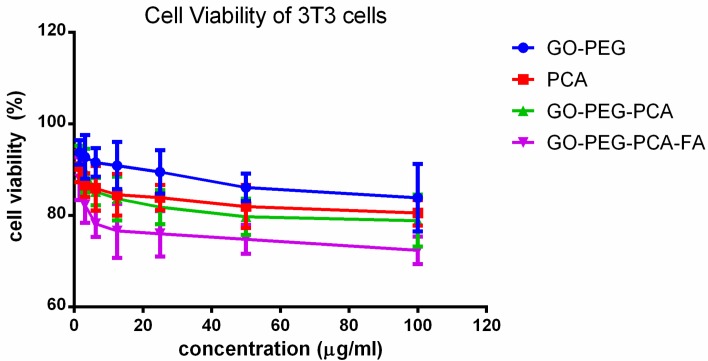
Cell viability of normal fibroblast (3T3) cell lines exposed to various gradient concentrations of the drug, PCA, and their nanoparticles delivery systems, GO–PEG, GO–PEG–PCA, and GO–PEG–PCA–FA. The data presented are the mean ± SD of triplicate values.

Cytotoxicity studies were conducted by treating free drug PCA, empty nanocarriers (GO–PEG), the anticancer nanocomposite (GO–PEG–PCA), and the folic acid-coated anticancer nanocomposite (GO–PEG–PCA) with normal fibroblast (3T3) cells. Various gradient concentrations of the samples were incubated for a maximum of 72 h with the 3T3 cells. Cell viability was determined using the standard MTT assay protocol [12,17,58,59]. Figure 3 shows the percentage cell viability of the 3T3 cells after 72 h of incubation with all of the samples. All of the samples, including free drug PCA, empty nanocarrier (GO–PEG), anticancer nanocomposite (GO–PEG–PCA), and the folic acid-coated anticancer nanocomposite (GO–PEG–PCA–FA) were found to be biocompatible and non-toxic, as the cell viability was found to be more than 80% after 72 h of incubation. This suggests that the designed anticancer nanocomposite formulation is biocompatible with normal cells, and would be very useful for targeting the cancer cells without damaging/harming the normal tissues. The ANOVA statistics revealed that no significant difference was found among the samples groups at individual concentrations using ANOVA and Duncan’s multiple range test.

### 2.8. Anticancer Action against Liver Cancer Cells, HepG2 

**Figure 4:**
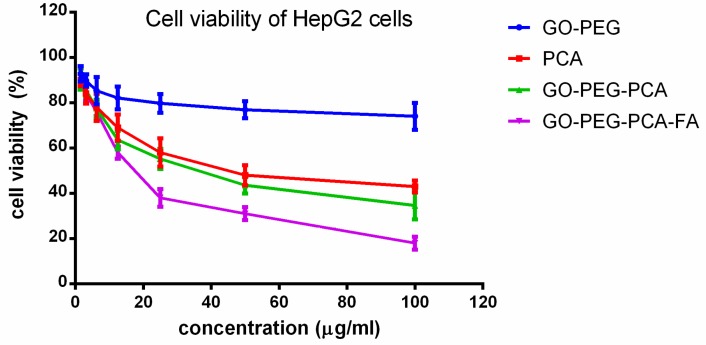
Viability of human liver cancer, HepG2, cell lines exposed to various gradient concentrations of the drug, PCA, and their nanocomposite delivery systems, GO–PEG, GO–PEG–PCA, and GO–PEG–PCA–FA. The data presented are the mean ± SD of triplicate values.

For the anticancer activity, the free drug PCA, empty nanocarrier (GO–PEG), anticancer nanocomposite (GO–PEG–PCA), and folic acid-coated anticancer (GO–PEG–PCA–FA) nanocomposite were treated with liver cancer cells, HepG2. Different concentrations of the above samples were incubated with liver cancer cells HepG2 for 72 h, and cell viability was determined by the MTT assay protocol. The empty carrier, GO–PEG, did not show any inhibitory action against liver cancer cells, HepG2. The IC_50_ of the pure drug PCA against liver cancer cells HepG2 was found to be 37.48 µg/mL. The IC_50_ of the (GO–PEG–PCA) and the folic acid-coated anticancer (GO–PEG–PCA–FA) nanocomposite were found to be 29.84 µg/mL and 18.89 µg/mL, respectively. The effective IC_50_, which is the actual amount of PCA present in 29.84 µg/mL and 18.89 µg/mL of IC_50_ before and after folic acid coating of the anticancer nanocomposite, and was calculated from percentage drug (PCA) loading, was determined using HPLC analysis to be 36.08%. Therefore, based on the percentage of PCA loading, the effective IC_50_ for the anticancer (GO–PEG–PCA) and (GO–PEG–PCA–FA) nanocomposite was calculated to be 10.76 g/mL and 6.81 μg/mL, respectively. These effective IC_50_ of the (GO–PEG–PCA) and (GO–PEG–PCA–FA) are much lower than the IC_50_ of the free PCA (i.e., 37.48 μg/mL). These results suggest that the nanocomposites have much better anticancer activity compared to the free drug, PCA. Furthermore, folic acid targeting (GO–PEG–PCA–FA) was also found to improve the anticancer effects, as the IC_50_ value of 6.81 μg/mL is lower than 10.76 μg/mL for the non-folic acid-coated (GO–PEG–PCA) nanocomposite. Table 2 shows the IC_50_ values of all of the samples used against HepG2 cells. The improved anticancer effect of the folic acid-coated nanocomposite against liver cancer cells (HepG2) can be attributed to the folate receptors that are present on the surface of the cancer cells, which help with better internalization. Figure 4 shows the cell viability results of liver cancer cells, HepG2, when it was treated with different concentrations of the free drug PCA, empty nanocarrier (GO–PEG), anticancer (GO–PEG–PCA), and the folic acid-coated (GO–PEG–PCA–FA) nanoparticles. Statistics were determined using software SAS 9.3, and ANOVA and Duncan’s multiple range test were performed. The significant differences were found between the empty carrier (GO–PEG), free drug PCA, and the nanocomposites GO–PEG–PCA; however, PCA and GO–PEG–PCA were insignificantly different. The nanocomposite GO–PEG–PCA–FA was found to be significantly different from all of the other samples at a concentration of 6.25 μg/mL and 12.5 μg/mL with (*p* values of <0.0007). At concentrations of 25 μg/mL and 50 μg/mL, the samples of PCA and GO–PEG–PCA and GO–PEG–PCA–FA were significantly different from the empty carrier. The sample PCA and GO–PEG–PCA were insignificantly different from one another. The folic acid coated sample GO–PEG–PCA–FA was significantly different from PCA alone, and GO–PGE–PCA. The value was found to be <0.0001. At the concentration of 100 μg/mL, all of the samples were found to be significantly differently *p* value of (<0.0001) from one another.

### 2.9. Anticancer Action against the Colorectal (Colon) Cancer (HT29) Cells

**Figure 5:**
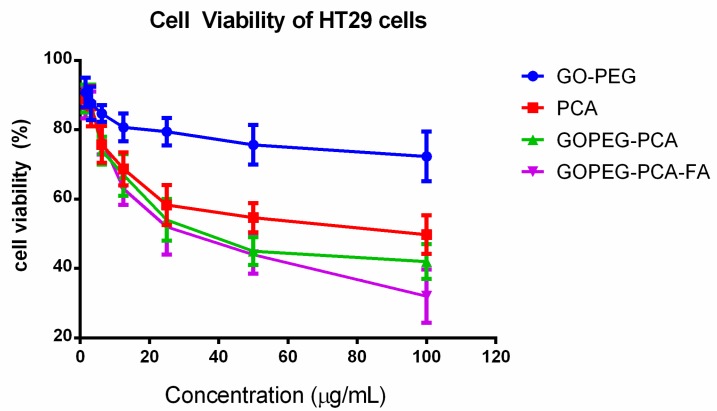
Cell viability of colon cancer (HT29) cell lines exposed to various gradient concentrations of the drug, PCA, and their nanoparticles delivery systems, GO–PEG, GO–PEG–PCA, and GO–PEG–PCA–FA. The data presented are the mean ± SD of triplicate values.

Figure 5, shows the anticancer effect of free drug PCA, empty nanocarrier (GO–PEG), anticancer (GO–PEG–PCA), and the folic acid-coated anticancer (GO–PEG–PCA–FA) nanocomposite against colorectal (colon) cancer (HT29) cells. The MTT protocol was used for cancer cell viability determination after the treatment, and 72 h incubation with the above-mentioned samples. The empty carrier, (GO–PEG), did not show any cytotoxic effect on the colorectal (colon) cancer (HT29) cells. The IC_50_ for the free drug PCA, anticancer delivery agent (GO–PEG–PCA), and folic acid-coated (GO–PEG–PCA–FA) nanocomposite were found to be 45.67 µg/mL, 38.65 µg/mL, and 30.56 µg/mL, respectively. The effective IC_50_ for the anticancer (GO–PEG–PCA) and folic acid-coated (GO–PEG–PCA–FA) nanocomposites were determined to be 13.94 µg/mL and 11.02 µg/mL, which is much lower than the IC_50_ of the free drug PCA of 45.67 µg/mL. The improved anticancer effects of the nanocomposite can be attributed to the nanoscaled size of the nanoparticles and folic acid-targeting of the folic acid-coated nanoparticles, which is similar to findings from the HepG2 cell lines studies. Table 2 shows the IC_50_ values of all of the samples used against HT29 cells. For the cell viability assays, ANOVA statistical analysis and Duncan's multiple range test were applied for the comparison between the samples at individual concentrations. There was no significant difference found up to the concentration of 6.12 μg/mL among the compounds. At the concentration of 12.5 μg/mL, a significant difference was found between the empty carrier GO–PEG and the other three compounds with a *p* value of <0.0002, but no significant difference was observed among the remaining samples. At the concentration of 25 μg/mL, a significant difference was found among the samples with *p* values of <0.0001; all of the samples were significantly different from the empty carrier GO–PEG. The free drug PCA and GO–PEG–PCA were significantly different from the folic acid coated samples GO–PEG–PCA–FA. At the concentration of 50 μg/mL, all of the GO–PEG–PCA and GO–PEG–PCA–FA were found to be significantly different from the free drug PCA, and these samples were also significantly different from the empty carrier GO–PEG with a *p* value of <0.0001. At the highest concentration used, 100 μg/mL, the statistics revealed that all of the samples were significantly different from one another with a *p* value of <0.0001.

All of the above cytotoxicity assays were carried out in triplicate, and the standard deviations were calculated and are incorporated into the respective bar graphs. For the calculation of IC_50_, we put the *x* and *y*-axis values in a graph for analysis and converted the *x*-axis values (conc.) to their log values.

This was followed by nonlinear regression (curve fit) under *XY* analysis to obtain the *y* = *a*(*X*) + *b* equation from the regression line; then, the inhibition IC_50_ values were calculated.

## 3. Discussion

The folate receptors that are present on the cancer cell surface are overexpressed on the vast majority of cancer tissues, and contrarily, its expression is limited in healthy tissues and organs. Several studies suggested that the colorectal (Colon) cancer cell (HT29) and human liver cancer (HepG2) are overexpressed with folate receptors [60,61,62,63]. In this study, we have designed an anticancer nanocomposite formulation using graphene oxide fabricated with polyethylene glycol as the nanocarrier loaded with the anticancer drug protocatechuic acid (GO–PEG–PCA). This designed nanocomposite formulation was further coated with folic acid (GO–PEG–PCA–FA) for active targeting of the cancer cells. All of the samples—free drug PCA, empty nanocarrier GO–PEG, anticancer nanocomposite (GO–PEG–PCA) without FA coating, and the anticancer nanocomposite coated with folic acid (GO–PEG–PCA–FA), were physicochemically characterized in detail using XRD, FTIR, HR-TEM, DLS, HPLC, and UV/Vis spectrophotometer. XRD and FTIR confirmed the successful formation of the anticancer nanocomposite and percentage drug loading PCA was found to be 36.08% by HPLC analysis, which further complimented the XRD and FTIR results. The HR-TEM results revealed the particle type shape of the GO–PEG–PCA–FA with a narrow particle size distribution between 4–30 nm with an average particle size of 8.72 nm was determined using image processing software by randomly selecting the 121 particles (N = 121) from an HR-TEM micrograph. The particle size of the nanocomposite (GO–PEG–PCA–FA) was also determined by the zeta sizer in its aqueous solution form, and particle size distribution was found to be between 8–25 nm, with an average size of 15 nm. For the release studies, UV/Vis spectrometer was used, and the absorption of aliquot was recorded at different times to determine the release kinetics. The in vitro release of the PCA from the anticancer nanocomposite was found to be highly sustained in a human body simulated phosphate-buffered saline (PBS) solution of blood pH 7.4 and intracellular lysosomal pH 4.8 at a temperature of 37 °C. The in vitro release under both physiological conditions of pH 7.4 and pH 4.8 took about 140 h (5.5 days). The designed anticancer nanocomposite GO–PEG–PCA–FA and all of the other samples such as empty nanocarrier GO–PEG, free drug PCA, and nanocomposite without folic acid coating GO–PEG–PCA were found to be highly biocompatible with normal 3T3 cells. For the evaluation of anticancer effects, all of the samples were tested against colorectal (colon) cancer cells (HT29) and liver cancer cells (HepG2). The IC_50_ of free drug PCA against colorectal cancer cells was found to be 45.77 μg/mL; the IC_50_ of the anticancer nanocomposite without coating of folic acid, GO–PEG–PCA, was found to be 38.65 μg/mL; whereas the nanocomposite coated with folic acid (GO–PEG–PCA–FA) was found to be the IC_50_ value of 30.56 μg/mL. The effective IC_50_ anticancer nanocomposite without FA coating and with FA coating which is the actual amount of drug PCA present in them, were calculated to be 13.94 μg/mL and 11.02 μg/mL, respectively, as given in Table 2. The IC_50_ values against the human liver cancer (HepG2) cells of free drug PCA, anticancer nanocomposite without FA coating (GO–PEG–PCA), and with FA coating (GO–PEG–PCA–FA) were found to be 37.48 μg/mL, 29.84 μg/mL, and 18.80 μg/mL, respectively. This suggests the much better anticancer activity of the nanocomposites compared to the free drug PCA. Furthermore, the effective IC_50_ of nanocomposites GO–PEG–PCA and GO–PEG–PCA–FA against human liver cancer (HepG2) were calculated to be 10.76 μg/mL and 6.81 μg/mL respectively, which is a much lower concentration than the free drug PCA. Thus, it can be inferred that the designed nanocomposite has a much better anticancer effect than the free drug PCA. The improved anticancer effect can be attributed to the nanometer size of the nanocomposites, prolonged sustained release, and folic coating. 

## 4. Materials and Methods

### 4.1. Materials

Graphite flakes (109 meshes), sulfuric acid (H_2_SO_4_ 98%), phosphoric acid (H_3_PO_4_), potassium permanganate (KMnO_4_), hydrogen peroxide, and phosphate-buffered saline (PBS) were purchased from Sigma Aldrich (St. Louis, MO, USA) and utilized without further purification. Diethyl ether, sodium hydroxide, hydrochloric acid (HCl, 37%), and ethyl alcohol (99.7% *v*/*v*) were bought from Friedemann Schmidt (Parkwood, WA, USA). The cell lines used for cytotoxicity assays are 3T3 (fibroblast cell), HT29 (human colorectal adenocarcinoma), and HepG2 (human hepatocellular carcinoma). Deionized water was used in all of the experiments.

### 4.2. Synthesis

#### 4.2.1. Synthesis of Graphene Oxide and GO–PEG

Graphene oxide (GO) was synthesized by the improved Hummers method. In brief, concentrated H_2_SO_4_ (360 mL) was mixed with 40 mL of concentrated H_3_PO_4_ and added to a mixture of 3 g of graphite powder and 18 g of KMnO_4_. The solution was continuously stirred at 50 °C for 12 h. After that, the resultant suspension was poured on 400 g of ice cubes containing 3 mL of hydrogen peroxide, and then, the final solution was washed with 200 mL of deionized water, 200 mL of HCl, and 200 mL of ethanol. Finally, the sample was coagulated with diethyl ether and then dried at 40 °C [57]. For the synthesis of GO–PEG, the dried powder of the synthesized GO (1 g) was dispersed into 50 mL of 1% PEG aqueous solution with the pH being raised to basic, and then the sample was continuously stirred for 24 h. Then, the sample was centrifuged, washed thoroughly using ethanol, and dried at 40 °C.

#### 4.2.2. PCA Loading on GO–PEG and Folic Acid Coating

In 50 mL of 5% PCA solution, 1 g of GO–PEG was added, and the sample was stirred for 24 h. Then, the sample was washed thoroughly and dried in an oven at 40 °C. After that, the samples were ground to a powder, and the sample was added to 50 mL of 1% folic acid solution and continuously stirred for another 24 h followed by centrifugation, thorough washing, and drying in an oven at 40 °C. The resulting nanocomposite GO–PEG–PCA–FA was ground to a fine powder and subjected to further characterization.

#### 4.2.3. UV/Vis Spectrophotometric Analysis

Optical and kinetic studies were carried using Perkin Elmer UV/Vis spectrophotometer (Model Lambda 35). The lambda max for PCA was determined to be 256 nm, and this wavelength was used for in vitro release kinetic studies. For in vitro release studies, approximately 3 mg of PCA nanocomposite was put in 3 mL of buffer of pH 7.4 and pH 4.8 separately, and the release profile was determined by taking absorption at different time intervals. 

#### 4.2.4. HPLC Analysis

Waters HPLC was used for the quantification of PCA in the nanocomposite. In brief, the instrument that was used was Waters HPLC model 2695 equipped with an Agilent C18 column (5 μm particle size; 4.6 × 250 mm), photodiode array (PDA) detector, and Empower software (Waters, Milford, MA, USA). The mobile phase consisted of acetonitrile water (adjusted to pH 3 with H_3_PO_4_, 9:91, *v*/*v*) and the flow rate was kept at 1 mL/min and hydroxybenzoic acid was used as an internal standard. 

#### 4.2.5. Physicochemical Characterization

X-ray diffraction (XRD) patterns were recorded by using the CuK_α_ radiation (λ = 1.5418 Å) at 30 kV and 30 mA with an XRD-6000 Diffractometer, Shimadzu, Tokyo, Japan). A Perkin Elmer ultraviolet-visible spectrophotometer, model Lambda 35, was utilized for the quantification of drug loading and in vitro release properties. The high-resolution transmission electron microscope (HR-TEM), model Technai G2 (FEI Company, Hillsboro, OR, USA) was used for the surface and morphological properties. For the Raman analysis, a Raman spectrometer (Model Alpha 300R Witec, GmbH Lise-Meitner-Straße 6 D-89081 Ulm, Germany) with an excitation wavelength at 532 nm was used in this study. Functional groups were recorded in the range of 500–4000 cm^−1^ analyzed by a Fourier transform infrared (FTIR) of Perkin-Elmer 100 series ((PerkinElmer, Inc. Waltham, MA, USA) by a direct sample method. Dynamic light-scattering technique, using Zeta sizer nanoseries —NANO-S Malvern instrument (WR14 1XZ, Worcestershire, UK) were used for the particle size distribution analysis of the anticancer nanoparticles GO–PEG–PCA–FA, using a Zetasizer (Malvern Instruments Model: Nano S). Optical and kinetic studies were carried out using Perkin Elmer UV/Vis spectrophotometer Model Lambda 35 (PerkinElmer, Inc. Waltham, MA USA). Statistical analyses were performed using software SAS 9.3 (SAS, Cary, NC, USA).

#### 4.2.6. Cell Culture and MTT Cell Viability Assays

In cell viability assays, normal cell such as 3T3 cell and cancerous cells including HepG2 and HT29 cell were acquired from Roswell Park Memorial Institute (RPMI) of 1640 medium supplemented with 10% fetal bovine albumin, penicillin, and streptomycin, and equilibrated with 5% carbon dioxide at 37 °C. A methylthiazol tetrazolium (MTT)-based assay was carried out to determine the cell viability. Cells were harvested and seeded in 96-well tissue culture plates at 1.0 × 10^4^ cells/well for 24 h. PCA, GO–PEG, and GO–PEG–PCA–FA nanoparticles stock solutions were prepared by dissolving the compound in 1:1 of dimethyl sulfoxide (0.1%) and RPMI. Then, the mixture was further diluted in the same media to produce various final concentrations, ranging from 1.25 μg/mL to 100 μg/mL. Once the cells were attached to the respective wells after 24 h, the tested compounds were added until the final volume of 100 μL/well were obtained. After 72 h of incubation, 10 μL of MTT solution (5 mg/mL in PBS) was added in each well and further incubated for 3 h before being aspirated. Then, 100 μL of dimethyl sulfoxide was added per well in order to dissolve the purple formazan salt. The intensity of the purple formazan solution, which reflects cell growth, was subsequently measured at 570 nm using a microplate reader.

## 5. Conclusions

In this study, an anticancer nanocomposite formulation was designed using protocatechuic acid (PCA) as an anticancer active agent and GO–PEG as the nanocarrier. For cancer targeting, the folic acid coating was employed on the nanoparticles, resulting in GO–PEG–PCA–FA. The in vitro release of the PCA from the designed anticancer nanocomposite was found to be highly sustained, and the release of PCA was found to be extended to about 125 h. The designed anticancer nanocomposites were found to show low toxicity to normal fibroblast cells (3T3). The effective IC_50_ of the designed anticancer nanocomposites against liver cancer, HepG2 cells, and colon cancer HT29 cells were found to be much lower than the free drug, PCA. In addition to this, the folic acid coating further lowered the IC_50_ against these cells lines. The statistical analysis of MTT assay against cancer cells revealed that a significant difference was found between the folic acid-coated nanocomposite GO–PEG–PCA–FA compared to free drug PCA and nanocomposite without folic acid coating. The improved efficacy can be attributed to the nanosize regime of the resulting nanocomposites with sustained release properties together with the presence of the folic acid targeting agent. These in vitro studies results are highly encouraging for further in vivo evaluation studies.

## Figures and Tables

**Table 1:** FTIR bands of functional groups of free drug PCA, FA, nanocomposite GO–PEG–PCA, and folic acid-coated nanocomposite (GO–PEG–PCA–FA).

Assignment	PCA	FA	GO–PEG–PCA	GO–PEG–PCA–FA
**N–H**	-	3542, 3460	-	3540, 3400
**O–H stretching**	3253	3319, 3414	3223	3318
**C–H aromatic stretching and PEG stretching**	3072	3091, 2927	2919, 2861	3110, 2922, 2869
**C=O**	1662	-	1620	1692
**C=O amide**	-	1688	-	1605
**C=N**	-	1636	-	1634
**Aromatic C–C stretching**	1500–1300	-	1500–1300	1500–1300
**C–O–C ether**	-	-	1071	1068
**C–H in plane bending of aromatic ring**	1040	-	1000	9043
**C–H bending** **N–H rocking**	760	762	703	761

**Table 2:** The half maximum inhibitory concentration (IC_50_) value for GO–PEG, PCA, GO–PEG–PCA, and GO–PEG–PCA–FA nanoparticles tested on 3T3, HT29, and HepG2 cell lines. The unit of IC_50 _in give Table 2 is μg/mL.

Type Cells	GO–PEG	PCA	IC_50_ GO–PEG–PCA	IC_50_ GO–PEG–PCA–FA	Effective IC_50_ GO–PEG–PCA	Effective IC_50_ GO–PEG–PCA–FA
**Fibroblast cell (3T3)**	NCT	NCT	NCT	NCT	NCT	NCT
**colorectal (** **Colon) cancer cell** ** (** **HT29)**	NCT	45.67	38.65	30.56	13.94	11.02
**Human liver cancer (HepG2)**	NCT	37.48	29.84	18.89	10.76	6.81

NCT = no cytotoxicity effect, PCA = Protocatechuic acid.
